# Supplementary material for: Complexome profiling of the Chlamydomonas psb28 mutant reveals TEF5 as an early PSII assembly factor
Source: Plant Cell. 2025 Mar 17;37(6):koaf055. doi: 10.1093/plcell/koaf055 (PMC12164590; doi:10.1093/plcell/koaf055)
Supplement: koaf055_Supplementary_Data [file koaf055_supplementary_data.zip › psb28_interactive.zip!/plots/intensity/qualityPlots/peptides/LHCBM9_Cre06.g284200.t1.2.html]

### Cre06.g284200.t1.2

Trivial name: LHCBM9  
SeqCoverage [median]: 37.80  
Mass: 27.07 kDa  
  
 

| Sequence | MissedCleavage | Mass [Da] | wtIntensity | muIntensity | Score |
| --- | --- | --- | --- | --- | --- |
| GAGIEFYGPNR | 0 | 1179.57 | 14931.11 | 1884.73 | 139.88 |
|  |  |  | 14931.1 | 1884.7 |  |
